# Effect of lymph node examined count on accurate staging and survival of resected esophageal cancer

**DOI:** 10.1111/1759-7714.13056

**Published:** 2019-04-07

**Authors:** Wenjie Xia, Suyao Liu, Qixing Mao, Bing Chen, Weidong Ma, Gaochao Dong, Lin Xu, Feng Jiang

**Affiliations:** ^1^ Department of Thoracic Surgery Nanjing Medical University Affiliated Cancer Hospital Nanjing China; ^2^ Jiangsu Key Laboratory of Molecular and Translational Cancer Research Cancer Institute of Jiangsu Province Nanjing China; ^3^ The Fourth Clinical College of Nanjing Medical University Nanjing China; ^4^ Department of Hematology and Oncology, Geriatric Lung Cancer Research Laboratory Jiangsu Province Geriatric Hospital Nanjing China

**Keywords:** Accurate staging, esophageal cancer, LNE, surgery, survival

## Abstract

**Background:**

We examined the association between numbers of lymph nodes examined (LNEs) and accurate staging and survival to determine the optimal LNE count during esophagectomy using data from the Surveillance, Epidemiology, and End Results (SEER) cancer registry and the Department of Thoracic Surgery of a single institution (SI).

**Methods:**

A total of 7356 EC patients met our inclusion criteria from the SEER database and 1275 patients from SI. We applied multivariate models to investigate the relationship between the LNE count and LN metastasis and cancer‐specific survival (CSS). Odds ratios (ORs) and hazard ratios (HRs) generated by the multivariate models were fitted with Locally Weighted Scatterplot Smoothing, and the structural breakpoints were determined by the Chow test.

**Results:**

Higher numbers of LNEs were linked to a higher proportion of LN metastasis and better CSS in both cohorts. Cut‐point analysis determined a threshold of LNEs of 12 for adenocarcinoma and 14 for esophageal squamous cell cancer (ESCC) considering accurate staging, and 15 for adenocarcinoma and 14 for ESCC considering OS. The cut‐points for CSS were examined in the SEER database and validated in the divided cohort from SI (all *P* < 0.05).

**Conclusion:**

A greater number of LNEs are significantly associated with more accurate N staging and better survival in EC patients. We recommend 15 and 14 as the threshold LNE counts for adenocarcinoma and ESCC patients, respectively.

## Introduction

Esophageal cancer (EC) is the sixth most common cause of cancer death globally and the incidence of esophageal adenocarcinoma is increasing. Prognosis is poor, with a five‐year survival rate of < 15%.^1^, ^2^ Esophagectomy is the mainstay therapy for EC patients without systemic metastases. The pathologic status of the regional nodes significantly influences recurrence rates and survival after surgery. During esophagectomy, lymph node (LN) sampling or dissection plays an important role in precise nodal staging by identifying LN involvement and determining the extent of disease and the therapeutic effect of potential LN metastatic lesion clearance.

However, the optimal number of lymph nodes examined (LNEs) during surgery is controversial and requires clarification.^3^, ^4^, ^5^, ^6^ Although extended lymphadenectomy seems to improve survival and is considered the criterion standard in many clinical guidelines, it has been suggested that a more extensive lymphadenectomy confers no advantage with respect to survival but does have the disadvantage of greater surgical morbidity.^7^, ^8^, ^9^ In addition to the survival effect, the impact of the LNE number on accurate staging in EC has not previously been evaluated.

In the present study, we applied data from two large databases including various regions, ethnicities, and clinical preferences, which may more accurately represent real‐world conditions to further confirm the relationship between LNEs and long‐term survival and staging to determine an optimal LNE count threshold.

## Methods

### Patient population

We conducted a retrospective analysis of two cohorts of patients. One cohort comprised EC patients who underwent esophagectomy with R0 resection at Nanjing Medical University Affiliated Cancer Hospital (single institution [SI]). The other cohort was obtained from the Surveillance, Epidemiology, and End Results (SEER) cancer registry. The Nanjing Medical University Review Board approved the research. The study is registered in the Chinese Clinical Trial Registry (ChiCTR1800018237).

#### Surveillance, Epidemiology, and End Results database

A total of 7356 patients with EC diagnosed from 2004 to 2008 were identified in the SEER database. The SEER program currently collects and publishes cancer incidence and survival data from population‐based cancer registries covering approximately 30% of the population of the United States.^10^ Eligible cases were identified as patients: with tumors with malignant behavior located in the esophagus as defined by the International Classification of Diseases for Oncology (ICD‐O‐3)/World Health Organization 2008 site code C150–C159; and who underwent esophagectomy with R0 resection. Patients diagnosed before 2004 were excluded because there was no specific American Joint Committee on Cancer 7th edition staging information prior to 2004, while patients diagnosed after 2008 were excluded to ensure that we had adequate follow‐up data to evaluate five‐year mortality. We also excluded patients who underwent local excision or local destruction, because an examination of LNs is not standard in this procedure. Patients without detailed information of tumor node metastasis (TNM) stage and pathological type were excluded. Given that a large proportion of EC patients receive adjuvant or neoadjuvant therapy, we did not exclude these patients and controlled for this factor in multivariate analysis.^11^, ^12^

#### Patients from the single institution

Clinical, pathological, and follow‐up information from the SI was collected with the assistance of the Large‐scale Data Analysis Center of Cancer Precision Medicine‐LinkDoc database. A total of 1275 EC patients were recruited from 2013 to 2016. Written informed consent was obtained from all participants involved in this study. The main surgical approach was Ivor Lewis esophagectomy; however, a few patients were also treated via three‐way or left esophagectomy. LNs were harvested during surgical resection and pathologists examined the tissues postoperatively. Patients without detailed information of TNM stage or pathological type were excluded. As most of the patients were not fully followed‐up for five years, SI patient data were used to build the logistic regression analysis to examine the effect of LNE on LN metastasis; however, the data were not analyzed by Cox multivariate analysis. Furthermore, an independent cohort (330 patients) that comprised EC patients who were followed‐up for five years was also used for cutoff validation.

### Statistical analyses

#### Multivariate regression analyses

On the basis of the theory that more LNEs present a greater opportunity to identify positive LNs, LN metastasis was assessed by correlating the number of LNEs and the proportion of each N stage category (node negative vs. node positive) using a binary logistic regression model after adjusting for age, gender, T stage, histologic grade, histologic type, administration of neoadjuvant therapy, tumor location, and surgical procedure. We also performed stratified analyses by T stage and histologic type. Cox regression models were used to evaluate the association between the number of negative LNEs and EC‐specific survival after adjusting for potential confounders, such as gender, age, T stage, histologic grade, histologic type, tumor location, administration of neoadjuvant or adjuvant therapy, and LN metastasis. We also performed stratified analyses by T stage, histologic type, and LN metastasis. Multivariate regression analyses were performed using SPSS version 19.0.

#### Threshold of the number of lymph nodes

**Curves were generated using odds ratios (ORs, each LNE count compared to 1 LNE as a reference) in logistic regression analysis and hazard ratios (HRs) in Cox regression analysis using Locally Weighted Scatterplot Smoothing (LOWESS, Stata 12.0) with a bandwidth of 0.6. Structural breakpoints were then determined by the Chow test using EViews 9.0 software and were considered the threshold of clinical impact.^13^ The Chow test is often used to determine whether independent variables have different impacts on different subgroups of the population.

#### Cutoff validation in the divided cohort

We used the unadjusted Kaplan–Meier method for visualization of the survival curves, and the log‐rank test to compare survival curves using SPSS version 19.0. Cox regression was also performed to examine the generated cutoff LNE number.

## Results

### Patient characteristics and distribution of lymph nodes examined (LNEs)

**Figure 1:**
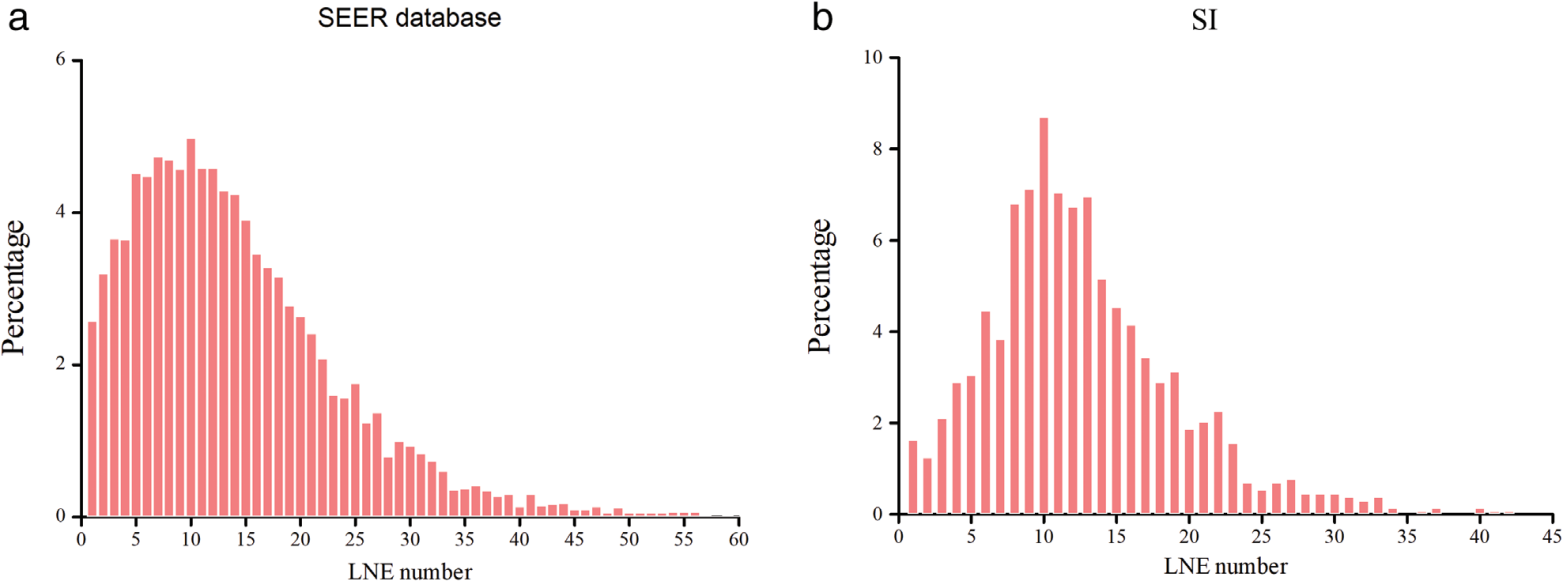
Distribution of the number of lymph nodes examined (LNE) in patients with esophageal cancer (EC) from the (**a**) Surveillance, Epidemiology, and End Results (SEER) database and the (**b**) Thoracic Surgery Department of a single institution (SI).

The SEER database and SI cohorts included 7356 and 1275 EC patients, respectively. Among the SEER cohort, there were 5664 (76.7%) adenocarcinoma patients and 1558 (15.2%) esophageal squamous cell carcinoma (ESCC) patients. The proportion of ESCC patients in the SI cohort was too small to permit statistical analysis, thus ESCC and adenocarcinoma were combined. The baseline characteristics are listed in Table 1. The median number of LNEs was 12 for both cohorts. The distributions are presented in Figure 1. The median survival of the SEER cohort was 36 months (95% confidence interval [CI] 33.99–38.01). Because only 330 SI patients were followed‐up to 24 months, they were separated as a single cohort to validate the cutoff point generated by analysis of the SEER survival data.

**Table 1:** Demographics and tumor characteristics of patients with esophageal cancer

Variable	SEER database, N (%)	Single institution, N (%)
Age		
< 50	636 (8.6)	49 (3.8)
50–59	1860 (25.3)	280 (22.0)
60–69	2817 (38.3)	724 (56.8)
≥ 70	2043 (27.8)	222 (17.4)
Gender		
Male	6155 (83.7)	1025 (80.4)
Female	1201 (16.3)	250 (19.6)
Histologic grade		
G1	509 (6.9)	209 (16.4)
G2	2837 (38.6)	523 (41.0)
G3	3157 (42.9)	322 (25.3)
G4	113 (1.5)	151 (11.8)
Unknown	740 (10.1)	70 (5.5)
Histology		
ESCC	1558 (21.2)	1275
Adenocarcinoma	5644 (76.7)	—
Other	154 (2.1)	—
T stage		
T1	2094 (28.5)	304 (23.8)
T2	1179 (16.0)	325 (25.5)
T3	3652 (49.6)	337 (26.4)
T4	431 (5.9)	309 (24.2)
N stage		
N0	4596 (62.5)	763 (59.8)
N1	2760 (37.5)	512 (40.2)
Surgical approach		
Ivor Lewis	N/A	871 (68.3)
Three‐way	N/A	217 (17.2)
Left	N/A	187 (14.7)
Tumor location		
Upper third	377 (5.1)	116 (9.10)
Middle third	1099 (14.9)	519 (40.7)
Lower third and esophagogastric junction	5880 (79.9)	640 (50.2)
Number of nodes resected		
1–10	3027 (41.2)	519 (40.7)
11–20	2723 (37.0)	587 (46.0)
21–30	1093 (14.9)	128 (10.0)
> 30	513 (7.0)	21 (1.6)
Number of nodes positive		
0	4596 (62.5)	770 (60.4)
1–2	1556 (21.2)	316 (24.8)
3–6	791 (10.8)	157 (12.3)
> 7	413 (5.6)	32 (2.5)
Median LNE count (IQR)	12 (7–19)	12 (8–16)

ESCC, esophageal squamous cell carcinoma; IQR, interquartile range; LNE, lymph nodes examined; N/A, not available; SEER, Surveillance, Epidemiology, and End Results.

### Impact of LNE counts on accurate N staging

It is conceivable that more LNEs present a greater opportunity to identify positive LNs, as well as more accurate N staging. Univariate and multivariate logistic analyses were performed to evaluate this postulation (Table 2). After controlling for age, gender, T stage, histologic grade, histologic type, administration of neoadjuvant therapy, tumor location, and surgical procedure, higher LNE counts were significantly associated with an increased proportion of LN metastasis in both cohorts (SEER: OR 1.021, 95% CI 1.016–1.025, *P* < 0.001; SI: OR 1.045, 95% CI 1.026–1.065, *P* < 0.001). In stratified analysis by T stage and histologic type, the differences remained significant in most subgroups, except for stage T2 SI patients.

**Table 2:** Univariate and multivariate analyses of LNE on LN metastasis

Subgroup	Univariate analysis		Multivariate analysis	
	OR (95% CI)	*P*	OR (95% CI)	*P*
SEER summary	1.021 (1.017–1.026)	< 0.001	1.021 (1.016–1.025)	< 0.001
Histologic type				
ESCC	1.013 (1.004–1.023)	0.004	1.014 (1.005–1.024)	0.003
Adenocarcinoma	1.024 (1.019–1.030)	< 0.001	1.023 (1.017–1.029)	< 0.001
Other types	1.028 (0.991–1.067)	0.145	1.019 (0.978–1.061)	0.374
T stage				
T1	1.019 (1.008–1.030)	< 0.001	1.020 (1.009–1.031)	< 0.001
T2	1.018 (1.006–1.029)	0.002	1.018 (1.007–1.030)	0.002
T3	1.021 (1.014–1.028)	< 0.001	1.020 (1.014–1.027)	< 0.001
T4	1.025 (1.008–1.043)	0.005	1.027 (1.010–1.045)	0.002
Single institution summary	1.048(1.030–1.067)	< 0.001	1.045(1.026–1.065)	< 0.001
T stage				
T1	1.102(1.051–1.155)	< 0.001	1.103(1.049–1.160)	< 0.001
T2	1.000(0.966–1.035)	0.997	1.001(0.965–1.038)	0.961
T3	1.053(1.018–1.089)	0.003	1.055(1.019–1.092)	0.002
T4	1.040(1.004–1.077)	0.027	1.404(1.004–1.077)	0.029

CI, confidence interval; ESCC, esophageal squamous cell carcinoma; LNE, lymph nodes examined; OR, odds ratio; SEER, Surveillance, Epidemiology, and End Results.

### Impact of LNE counts on survival

Variables entered into the Cox regression analysis included gender, age, T stage, histologic grade, histologic type, tumor location, administration of neoadjuvant or adjuvant therapy, and LN metastasis. More LNEs were significantly associated with better survival in patients with or without LN metastasis (N negative patients: HR 0.982, 95% CI 0.977–0.987, *P* < 0.001; N positive patients: HR 0.989, 95% CI 0.984–0.993, *P* < 0.001) (Table 3). After stratification by histologic type and T stage, the differences remained significant in most subgroups.

**Table 3:** Cox regression analysis of LNE on OS

Subgroup	OS (N0 disease)		OS (N+ disease)	
	HR (95% CI)	*P*	HR (95% CI)	*P*
SEER summary	0.982 (0.977–0.987)	< 0.001	0.989 (0.984–0.993)	< 0.001
Histologic type				
ESCC	0.985 (0.977–0.994)	0.001	0.990 (0.980–0.999)	0.037
Adenocarcinoma	0.981 (0.975–0.988)	< 0.001	0.988 (0.983–0.994)	< 0.001
Other types	0.962 (0.919–1.008)	0.102	0.971 (0.929–1.014)	0.186
T stage				
T1	0.989 (0.979–0.998)	0.020	0.981 (0.966–0.997)	0.022
T2	0.978 (0.966–0.990)	< 0.001	0.982 (0.969–0.995)	0.006
T3	0.981 (0.974–0.989)	< 0.001	0.989 (0.983–0.995)	< 0.001
T4	0.974 (0.955–0.993)	0.007	1.000 (0.988–1.013)	0.964

CI, confidence interval; ESCC, esophageal squamous cell carcinoma; LNE, lymph nodes examined; HR, hazard ratio; N0, node negative; N+, node positive; OS, overall survival; SEER, Surveillance, Epidemiology, and End Results.

### Cut‐point analyses and validation

**Figure 2:**
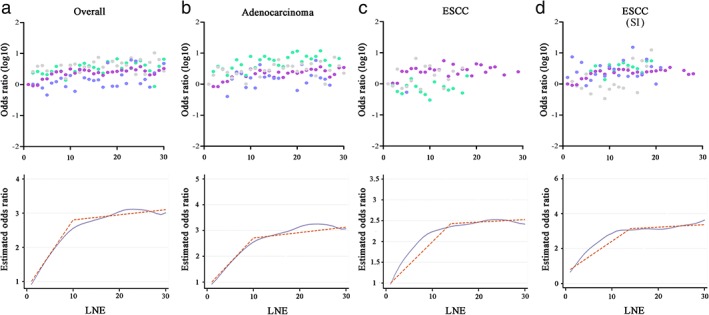
Co‐plot of odds ratios (ORs, upper row) and Locally Weighted Scatterplot Smoothing curves of stage migration and determination of structural break points with the use of the Chow test (bottom row). The fitting bandwidth was 0.6. Each dot in the co‐plot represents an OR of a specific lymph node examined (LNE, vacant if case number < 10) from logistic regression analysis. (**a**) Overall patients, (**b**) adenocarcinoma patients, and (**c**) esophageal squamous cell carcinoma (ESCC) patients from the Surveillance, Epidemiology, and End Results (SEER) database; and (**d**) ESCC patients from the Thoracic Surgery Department of a single institution (SI). (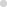) T1, (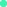) T2, (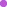) T3, and (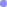) T4.

To determine the number of nodes that needed to be removed to obtain the maximum differential for accurate N staging and survival, ORs and HRs (each LNE count was compared to 1 LNE as a reference) were extracted from the logistic and Cox regression analyses to construct LOWESS smoother fitting curves and to calculate the structural breakpoint. A co‐plot of ORs and HRs revealed a similar trend as previous observations in that higher LNE counts were significantly associated with an increased proportion of LN metastasis and better CSS among most subgroups. In regard to N staging, the ORs of the structural breakpoints were 10, 12, and 14 for overall, adenocarcinoma, and ESCC patients, respectively (Fig 2). The structural breakpoint generated for SI patients was also 14, exactly the same as ESCC patients from the SEER dataset.

**Figure 3:**
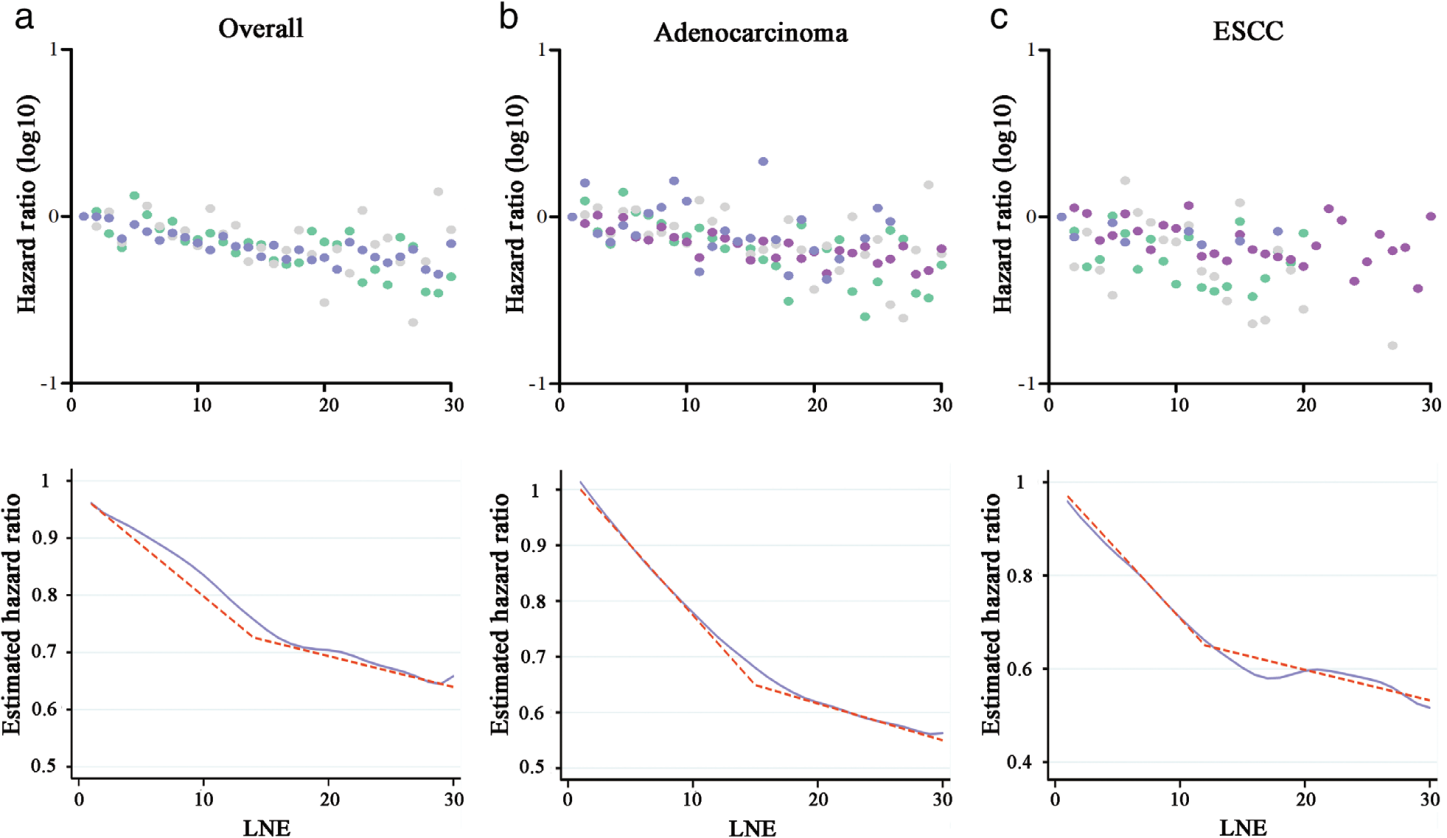
Co‐plot of hazard ratios (HRs, upper row) and Locally Weighted Scatterplot Smoothing curves of cancer‐specific survival (CSS) and determination of structural break points with use of the Chow test (bottom row). The fitting bandwidth was 0.6. Each dot in the co‐plot represents an HR of a specific lymph node examined (LNE, vacant if case number < 10) from Cox regression analysis. (**a**) Overall patients, (**b**) adenocarcinoma patients, and (**c**) esophageal squamous cell carcinoma (ESCC) patients from the Surveillance, Epidemiology, and End Results (SEER) database. (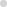) T1, (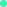) T2, (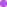) T3, and (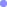) T4.

**Figure 4:**
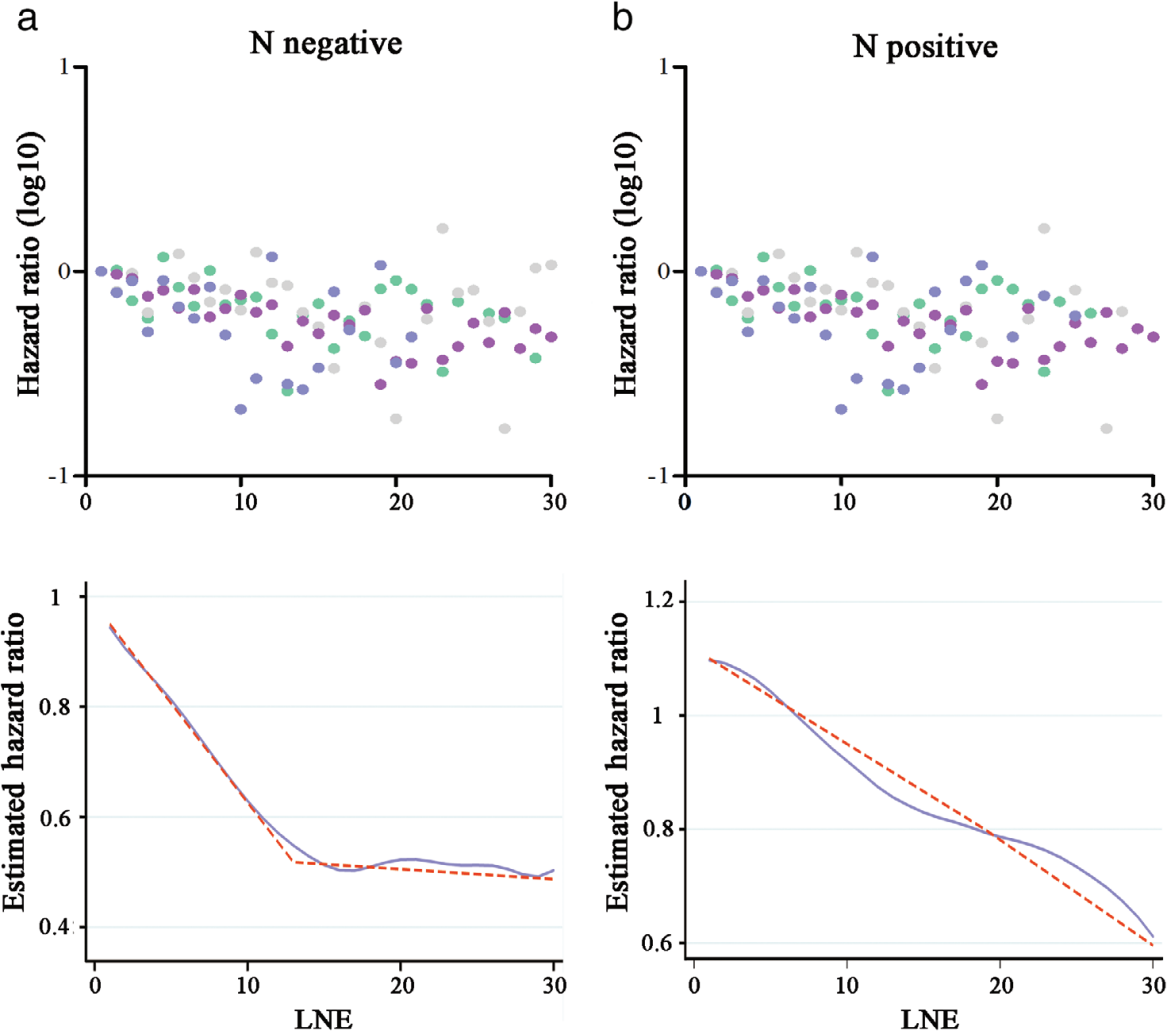
Co‐plot of hazard ratios (HRs, upper row) and Locally Weighted Scatterplot Smoothing curves of cancer‐specific survival (CSS) and determination of structural break points with the use of the Chow test (bottom row). The fitting bandwidth was 0.6. Each dot in the co‐plot represents an HR of a specific lymph node examined (LNE, vacant if case number < 10) from Cox regression analysis. (**a**) Node‐negative (N negative) and (**b**) node‐positive (N positive) patients. (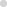) T1, (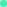) T2, (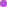) T3, and (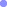) T4.

In regard to survival, after stratification by histologic type, the HRs of the structural breakpoints were 14, 15, and 12 for overall, adenocarcinoma, and ESCC patients, respectively (Fig 3). After stratification by LN metastasis, the HRs of the structural breakpoints were 13 and 19 for N negative and positive patients, respectively (Fig 4). Notably, for N positive patients, the Chow test of every count of LNE had a *P* value > 0.05 and a breakpoint was hardly identified; thus, the count of 19 was selected as a relatively suitable F value.

**Figure 5:**
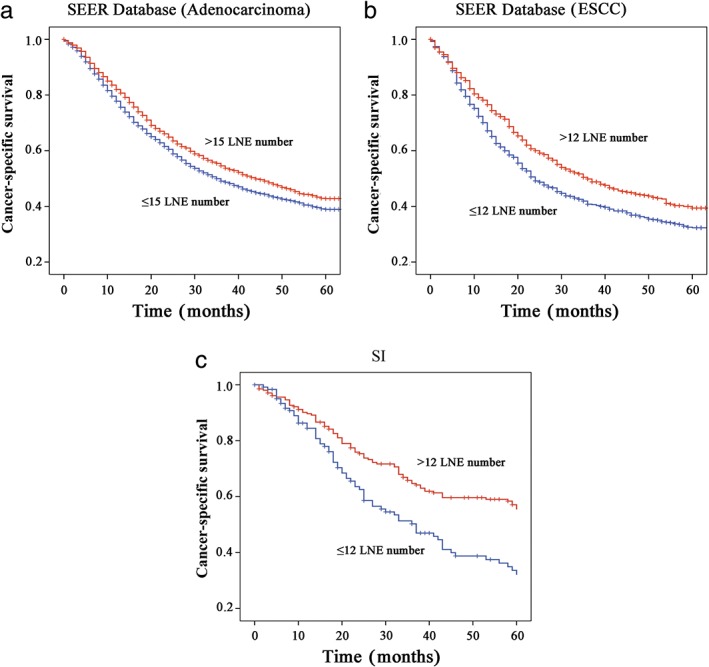
Cut‐point validation among (**a**) adenocarcinoma patients (cut‐point = 15), and (**b**) esophageal squamous cell carcinoma (ESCC) patients (cut‐point = 12) from the Surveillance, Epidemiology, and End Results (SEER) database and (**c**) ESCC patients from Thoracic Surgery Department of a single institution (SI, cut‐point = 12). LNE, lymph nodes examined.

HRs for the breakpoints (15 for adenocarcinoma, 12 for ESCC) were examined in the SEER cohort. In the adenocarcinoma subgroup, median survival was 35 months (95% CI 32.31–37.69) for patients with ≤ 15 LNE and 44 months (95% CI 39.71–48.30) for patients with >15 LNE (*P* < 0.001) (Fig 5a). In the ESCC subgroup, median survival was 24 months (95% CI 20.69–27.31) for patients with ≤ 12 LNE and 36 months (95% CI 29.39–42.61) for patients with > 12 LNE (*P* < 0.001) (Fig 5b). In Cox multivariate analysis, the number of LNEs was confirmed as an independent predictor of CSS (adenocarcinoma: HR 0.869, 95% CI 0.804–0.940, *P* < 0.001; ESCC: HR 0.785, 95% CI 0.686–0.899, *P* < 0.001). The break point (12 for ESCC) was then validated in the independent SI cohort (HR 0.573, 95% CI 0.348–0.914; *P* = 0.008) (Fig 5c).

## Discussion

The SEER and SI analyses revealed an independent association between LNE count and LN metastasis and survival in EC patients. Invariably, greater numbers of LNEs were linked to a higher proportion of LN metastasis and better CSS, which was observed in subgroups stratified by T stage, histologic type, and LN metastasis. According to the LOWESS smooth line and Chow test results, with regard to the accuracy of staging, we identified 12 and 14 as the minimum number of LNEs for adenocarcinoma and ESCC patients, respectively. With regard to survival, a minimum number of 15 and 12 patients should be examined to achieve optimal CSS for adenocarcinoma and ESCC, respectively. The threshold number of LNEs for CSS was examined in the SEER database and validated in a smaller cohort of patients from an SI. For comprehensive consideration, we recommend 15 and 14 as the threshold LNE counts for patients with adenocarcinoma and ESCC, respectively. Notably, among N positive patients, a threshold LNE count was difficult to determine through cut‐point analysis. Therefore, for patients who are suspected of being N positive based on preoperative imaging diagnosis, the LN should be resected as thoroughly as possible during surgery.

Surgery remains the fundamental modality for achieving esophageal cure.^14^ Surgeons have been exploring the role of more extensive resection and, in particular, the role of more extensive lymphadenectomy to improve survival rates.^7^, ^15^, ^16^, ^17^ Current clinical guidelines that advocate transthoracic esophagectomy with two‐field lymphadenectomy as the standard of care are being challenged.^8^, ^9^ Lagergren *et al.* examined the role of the extent of lymphadenectomy in relation to survival in patients with EC and reported that the total number of nodes removed was not associated with survival.^18^ Specifically, five‐year survival was the same in patients who had 21–52 nodes resected (fourth quartile) compared to patients who had 0–10 nodes removed (lowest quartile). Interestingly, this study replicated the results of a previously published report from Sweden that examined the effect of the number of resected LNs on survival among 1044 patients with EC. A more extensive lymphadenectomy was not associated with better survival.^19^

Clearly, these studies reported conflicting results. Thus, whether more extensive LN resection improves survival or rather just allows for better staging and migration remains unresolved. However, no previous research has focused on the impact of LNE count on accurate staging in EC patients.^9^, ^20^, ^21^, ^22^ We suggest that the LNE count during surgery should consider both accurate staging and better survival, regardless of the complex causality between them.

To our knowledge, this is the first study to comprehensively examine the relationship between LNE count and accurate N staging and survival in EC patients. The results demonstrate a significant association between the number of pathological LNEs at the time of esophagectomy with the proportion of LN metastasis patients. The therapeutic effect of a higher LNE count, which is even more meaningful when remnants are eliminated, indeed improves the survival of EC patients, especially given the invariable results in a subgroup of N negative patients. In addition, we applied the LOWESS smoother to generate the fitting curve and used the Chow test to determine the breakpoints as minimal clinical threshold LNE counts.^23^, ^24^, ^25^, ^26^ The Chow test is most commonly used to test for the presence of a structural break by estimating whether the independent variables have different impacts on different subgroups of the dependent variable.^13^

There are some limitations to the present study. As a retrospective study, we were not able to investigate some other important points, such as the administration of postoperative adjuvant chemoradiotherapy. However, we applied subgroup analysis (by T stage and LN metastasis) to reduce the impact of missing information. The number of LNEs may be a marker for the adequacy of surgical, pathological, and/or institutional care, all of which can be influenced by the approach to and the quality of the procedures performed in clinical practice, and thus will affect treatment outcomes.^27^, ^28^ This problem is inevitable when we use data from the SEER database. Furthermore, the number of retrieved LNs does not necessarily correlate with the extent of the lymphadenectomy.^29^ The extent of lymphadenectomy can only be fully evaluated if the location of these nodes can also be determined; therefore, an in‐depth study of lymphadenectomy is needed.

In summary, the results of this study demonstrate that a greater number of LNEs has a significant association with more accurate node staging and better survival in EC patients. For comprehensive consideration we recommend 15 and 14 as the threshold LNE count for patients with adenocarcinoma and ESCC, respectively. For patients suspected as being N positive on the basis of preoperative imaging, three‐field lymphadenectomy is recommended as the preferred surgical approach to ensure sufficient LNEs.

## Disclosure

No authors report any conflict of interest.
